# Domestic Pets in the United Arab Emirates as Reservoirs for Antibiotic-Resistant Bacteria: A Comprehensive Analysis of Extended-Spectrum Beta-Lactamase Producing *Escherichia coli* Prevalence and Risk Factors

**DOI:** 10.3390/ani13101587

**Published:** 2023-05-09

**Authors:** Ihab Habib, Khaja Mohteshamuddin, Mohamed-Yousif Ibrahim Mohamed, Glindya Bhagya Lakshmi, Afra Abdalla, Abdulla Bakhit Ali Alkaabi

**Affiliations:** 1 Veterinary Public Health Research Laboratory, Department of Veterinary Medicine, College of Agriculture and Veterinary Medicine, United Arab of Emirates University, Abu Dhabi 15551, United Arab Emirates; drkhaja707@uaeu.ac.ae (K.M.); mohamed-yousif-i@uaeu.ac.ae (M.-Y.I.M.); glindya_l@uaeu.ac.ae (G.B.L.); afra.abdalla@uaeu.ac.ae (A.A.); 201603764@uaeu.ac.ae (A.B.A.A.); 2 Department of Environmental Health, High Institute of Public Health, Alexandria University, Alexandria 5424041, Egypt

**Keywords:** ESBL, dogs, cats, antimicrobial resistance, Middle East

## Abstract

**Simple Summary:**

Direct and indirect exposure of antibiotics to pet animals has been shown to play a role in the transmission of antimicrobial-resistant bacteria. Our study from the United Arab Emirates found that a public health relevant antibiotic-resistant strain of *Escherichia coli* (*E. coli*) bacteria is present in fecal swabs sampled from many healthy pet cats and dogs. The strain, known as extended-spectrum β-lactamases resistant (ESBL-R) *E. coli*, is resistant to many commonly used antibiotics, making it difficult to treat if it causes an infection. We found that 23.65% of the animals tested had ESBL-R *E. coli*. The bacteria were most commonly found in pets that had access to water in ditches and puddles. The study also found that 91% of the bacteria samples were multidrug resistant, meaning that they were resistant to multiple types of antibiotics. The findings from this study call for veterinarians to develop surveillance programs to monitor ESBL-R *E. coli* in pets and to reduce the risk of transmission to humans and the environment.

**Abstract:**

Extended-spectrum β-lactamases resistant (ESBL-R) *Escherichia coli* (*E. coli*) has been reported from healthy and sick pets. However, data from Middle Eastern countries, including the United Arab Emirates (UAE), are minimal. This study provides the first evidence of ESBL-R *E. coli* carriage among pets in the UAE. A total of 148 rectal swabs were collected from domestic cats (*n* = 122) and dogs (*n* = 26) attending five animal clinics in the UAE. Samples were cultured directly onto selective agar, and suspected colonies were confirmed as ESBL-producing using phenotypic and molecular methods. Confirmed isolates were screened for their phenotypic resistance to twelve antimicrobial agents using the Kirby Bauer method. The owners of the pets completed a questionnaire at the time of sampling, and the data were used to identify risk factors. ESBL-R *E. coli* was detected in rectal swabs of 35 out of 148 animals (23.65%) (95% confidence interval [CI]: 17.06–31.32). Multivariable logistic regression analysis identified cats and dogs with access to water in ditches and puddles as 3.71 (*p*-value = 0.020) times more likely to be positive to ESBL-R *E. coli* than those without access to open water sources. Ciprofloxacin resistance was evident in 57.14% (44/77) of the ESBL-R *E. coli* isolates. The percentage of resistance to azithromycin and cefepime was 12.99% (10/77) and 48.05% (37/77), respectively. The *bla_CTX-M_* gene was detected in 82% of the PCR-screened isolates (*n* = 50). Multidrug resistance (MDR) phenotypes were evident in 91% (70/77) of the isolates. In conclusion, ESBL-R *E. coli* was detected at a noticeable rate among healthy pet cats and dogs in the UAE, and the majority are MDR to clinically important antimicrobials such as fluoroquinolones and 3rd and 4th generation cephalosporins. Our results call for strengthening antimicrobial stewardship among companion animal veterinarians in the UAE to reduce the potential transmission of ESBL-R *E. coli* between pets, humans, and urban environments.

## 1. Introduction

Antimicrobial resistance (AMR) is a growing global One Health challenge, with significant societal costs due to increased human mortality, morbidity, and use of health care resources [1]. Due to the limited options available for effective antimicrobial treatment against their infections in humans and animals, the World Health Organization (WHO) highlighted that *Enterobacterales* expressing extended-spectrum β-lactamases (ESBLs) were global priority pathogens [2]. ESBL enzymes enable bacteria to hydrolyze extended-spectrum cephalosporins, penicillins, and monobactams and require the use of β-lactamase inhibitors such as tazobactam and clavulanic acid [3]. Particularly, ESBL resistant (ESBL-R) *Escherichia coli* (*E. coli*) has been isolated from food, environment, livestock, wildlife, and companion animals and has been positively correlated with the extensive use of β-lactam antibiotics in veterinary settings [4]. The spread of AMR in pathogenic and commensal bacteria that are well adapted for colonization of both humans and animals represents a significant concern as shared environments provide a venue for the acquisition and spread of resistant strains from one host to another [1].

Direct and indirect exposure to pet animals has been shown to play a role in the transmission of antimicrobial-resistant bacteria. Household sharing or intimate companionship between pet animals and humans has been documented in several studies to increase the likelihood of transmitting and spillover of resistant bacteria between humans and their companion pets [5]. In the context of One Health AMR investigations, several studies have identified ESBL-R *E. coli* isolates from healthy and sick dogs and cats [6,7]. Moreover, the co-carriage of ESBL-R *E. coli* in dogs and humans from the same households has been demonstrated in some studies [8,9]. Identifying the risk factors associated with ESBL-R *E. coli* in pet dogs and cats can contribute to adopting efficient strategies to limit AMR spread.

The current evidence concerning the worldwide prevalence and characterization of ESBL-R *E. coli* in dogs and cats has been reviewed by different authors, highlighting that the data in Middle Eastern countries are minimal [10,11]. The United Arab Emirates (UAE) is home to one of the highest percentages of expatriates in the Middle East and the Arab world. Thousands of expatriates working and living in the UAE bring their pet companions or adopt them from local shelters; moreover, ownership of companion pets has become popular among locals in the UAE in recent years [12]. The UAE pet food market reached a value of USD 55 million in 2018, highlighting the evolution in pet ownership in the UAE context in recent years [12]. Nevertheless, some new health risks, such as the zoonotic transmission of AMR at the human–pets interface, have never been carefully assessed in the UAE context. The current frequency of AMR in companion dogs and cats remains unknown in the UAE, undermining the assessment of their potential role as an element of the One Health spread of AMR in the community.

As part of the first UAE study of AMR in companion animals, we aimed to estimate the prevalence of rectal carriage of ESBL-R *E. coli* in a convenient sample of healthy cats and dogs in the Abu Dhabi and Dubai emirates of the UAE. Then, the multiple resistance patterns of the recovered ESBL-R *E. coli* against twelve antimicrobials were determined. The owners of the cats and dogs completed a short questionnaire at the time of sampling, and the data were used to identify some potential risk factors associated with the carriage of ESBL-R *E. coli*.

## 2. Material and Methods

### 2.1. Recruitment and Sampling

The study was conducted from April to December 2022. Clinics were approached to participate voluntarily in the study, which was approved by the animal ethics committee of the United Arab Emirates University (Permit number: ERA_2022_8520). The inclusion criteria for the animals recruited in the study were as follows: (i) a complete absence of clinical signs of infectious diseases assessed by the veterinarian collecting the sample, and (ii) the animals had been based in the UAE (no travel history) for at least one year before the sampling date. Considering the former inclusion criteria, convenient sampling was adopted with a total of 148 rectal swabs collected from domestic cats (*n* = 122) and dogs (*n* = 26) attending five animal clinics in the urban side of the Emirates of Abu Dhabi and Dubai, UAE. Rectal swabs were collected from cats and dogs with the owners’ consent, and the sampling was done by either the attending nurse or the veterinarian. Rectal swab samples were stored at 4 °C in a transport medium (Amies Agar Gel Transport Swab (Thermo Scientific™, Oxoid, Basingstoke, UK) and then transported in a cool box to the Veterinary Public Health Research Laboratory of the United Arab Emirates University (UAE, Al-Ain) for examination. 

### 2.2. ESBL-R E. coli Isolation and Confirmation

Rectal swabs from cats and dogs were suspended by vertexing for 30 s in 5 mL of Buffered Pepton Water (Oxoid, UK), and 100 μL from the suspension were then plated directly onto tryptone bile x-glucuronide (TBX) medium (Oxoid, UK) containing ESBL agar supplement (HiCrome ESBL Agar Supplement (FD278), HiMedia, India; per vial for 500 mL medium; ceftazidime (1.5 mg), cefotaxime (1.5 mg), ceftriaxone (1.0 mg), aztreonam (1.0 mg), and fluconazole (5.0 mg)) to facilitate the target recovery of ESBL-R *E. coli* [13]. Plates were incubated aerobically for up to 48 h at 37 °C. For positive TBX plates, a colony that exhibited typical characteristics was selected, and whenever feasible, two sample colonies were chosen if they varied in colony morphology on TBX-ESBL agar supplement plates. Well isolated colonies from the positive plates were re-streaked for purification, and then stored intrypticase soy broth (Oxoid, UK) supplemented with 50% glycerol (HiMedia, Thane, India) at −80 °C. *E. coli* isolates recovered from TBX-ESBL agar supplement plates were suspected as potential ESBL producers and were confirmed by E-test (Ezy MIC™ (HiMedia, Thane, India)), as previously described [14].

### 2.3. Antimicrobial Susceptibility Testing ESBL-R E. coli and PCR Screening

For samples confirmed as ESBL-R *E.coli*, one or more isolates (if they varied in colony morphology) from each positive sample were selected for analysis by the Kirby Bauer method (all discs were supplied from Oxoid, UK). The clinical breakpoint values from the Clinical and Laboratory Standards Institute were employed to assess the antimicrobial susceptibility phenotypes [15]. Given the public health focus of this study, the CLSI human medicine guidelines were used. The following twelve antibiotics were evaluated: ciprofloxacin (CIP (5 μg)), chloramphenicol (C (30 μg)), gentamicin (CN (10 μg)), tetracycline (TE (30 μg)), cefotaxime (CTX (30 μg)), trimethoprim-sulfamethoxazole (SXT (25 μg)), ampicillin (AMP (10 μg)), azithromycin (AZM (15 μg)), ceftriaxone (CRO (30 μg)), cefoxitin (FOX (30 μg)), imipenem (IPM (10 μg)), and cefepime (FEP (30 μg)). To ensure quality control, *E. coli* ATCC 25922 was utilized as the reference strain for the susceptibility test. According to previous definitions [16], isolates were classified as MDR if they exhibited resistance to at least one agent in three or more antimicrobial categories using CLSI breakpoints.

Phenotypically confirmed ESBL-R *E. coli* per positive sample was further characterized by extracting DNA from pure cultures to identify the β-lactamase genes, mainly *bla*_CTX-M_, *bla*_TEM_, *bla*_SHV,_ and *bla*_OXA_. DNA was extracted using a commercial kit (Wizard^®^ Genomic DNA Purification Kit (Promega, Madison, WI, USA)). PCR amplification reactions were performed in a 25 µL containing 12.5 µL of Promega Go Taq™ Master Mixes, 0.2 M concentrations of each primer, and 2 µL of DNA template. PCR cycles and gel running parameters were the same as Fang et al. [17] described.

### 2.4. Statistical and Risk Factors Analyses for ESBL-R E. coli

A questionnaire consisting of 13 variables was administered to cat and dog owners at the time of rectal swab collection. The primary outcome in the statistical analysis of this study was whether or not (binary outcome) a rectal swab sample from a pet animal (either dog or cat) was positive for ESBL-producing *E. coli*. No specific signs of clinical diseases were included in the questionnaire, and absence of signs of illness was only based on the routine examination conducted by the attending veterinarian. Potential risk factors analyzed in this study included animal species, age, and sex. The other variables considered in the risk factors analysis were as follows: (i) frequent time per week that the animal spent in the house or outside; (ii) the number of other animals kept in the household; (iii) if the dog/cat stayed in a pension/animal shelter in the past year; (iv) if the dog/cat frequently came in contact with farm or livestock animals; (v) whether the dog/cat had access to open water sources, either for drinking or swimming, in the last six months; and (vi) the type of the food that the owner provided to the dog/cat. All owners signed a consent form confirming their understanding and acceptance of the study objectives; however, their enrolment was voluntary, and therefore, for some samples and variables, missing data were evident.

Univariate and multivariate logistic regression analyses were used to evaluate the effect of various factors on the presence or absence of ESBL-R *E. coli* carriage. Variables with a significant result from univariate logistic regression analysis (*p*-value < 0.2) were included in the final multivariate model and used to calculate the final odds ratio confidence intervals for the statistically significant variables. The adequacy of the fitted final logistic regression model was assessed using standard diagnostic procedures such as the classification table, the Hosmer and Lemeshow goodness-of-fit test, receiver operating characteristics (ROC) plots, and sensitivity/specificity plots [18]. All statistical analyses were performed using Stata 16.0 (Stata Corp., College Station, TX, USA).

## 3. Results

### 3.1. Prevalence of ESBL-R E. coli and Characteristics of Pet Animals Included in the Study

ESBL-R *E. coli* was detected in rectal swabs of 35 out of 148 animals (23.65%) (95% confidence interval [CI]: 17.06–31.32). The study sample included 122 cats and 26 dogs, and the detection rate of ESBL-R *E. coli* varied (however not significantly (*p*-value = 0.121) from 26.23% to 11.54% among cats and dogs, alternatively. As indicated in Table 1, the samples originated from five clinics, and despite the low detection rate of ESBL-R *E. coli* in the samples from Clinic C ((13.51% (5/37)), compared to Clinic A ((28.26% (13/46))—reference category), there was an overall non-significant effect concerning the clinics from which the samples were collected. Results in Table 1 also indicate no significant effect of the age and sex concerning the detection of ESBL-R *E. coli* in the study animals.

Among the pet owners who voluntarily completed the questionnaire, 45.3% (58/128) indicated that they always keep their cats and dogs inside the home. In contrast, 10.15% (13/128) indicated keeping their cats and dogs always outside the home, and the frequency of ESBL-R *E. coli* was significantly higher (*p*-value = 0.031) among rectal swabs collected from these animals compared with animals always kept inside the home (Table 1). As presented in Table 1, cats and dogs that had access to water in ditches and puddles had significantly higher rates of ESBL-R *E. coli* (8/18 (44.44%), *p*-value = 0.010) compared with those who had no access to open water sources. Concerning the type of food that the owners provided to their cats and dogs sampled in this study, animals that received a diet containing raw meat had higher frequency of ESBL-R *E. coli* ((40.00% (6/15)), despite being not statistically significant (*p*-value = 0.075) compared with animals provided with commercial dry food.

### 3.2. Risk Factors Associated with ESBL-R E. coli

The epidemiological data and the initial univariate analysis for the potential risk factors among the study animals are shown in Table 1. Six variables showed a *p*-value < 0.20 associated with the detection of ESBL-R *E. coli* based on the univariate analysis (Table 1) and were included in the final multivariable logistic regression analysis. However, after exercising backward elimination—where the results of the Wald test for individual parameters are examined and the least significant effect that does not meet the level for staying in the model is removed—only one risk factor variable was retained (Table 1). According to the final multivariable logistic regression analysis, cats and dogs that had access to water in ditches and puddles were 3.71 (95% CI, 1.6–13.9; *p*-value = 0.020) times more likely to be positive to ESBL-R *E. coli* compared with those had no access to open water sources (Table 1).

**Figure 1:**
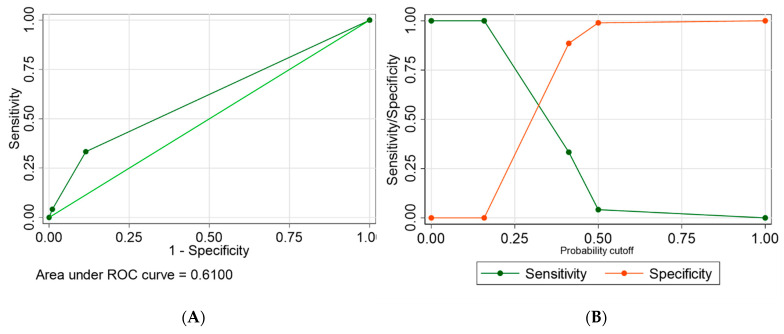
(**A**) A graph of receiver operating characteristics and calculation of the area under the curve, and (**B**) a graph of sensitivity and specificity as a function of the cut-off probability for the final multivariable logistic regression model used to fit risk factors associated with the positivity of rectal swab samples to ESBL-producing *E. coli* in dogs and cats in the UAE.

Figure 1A shows a receiver operating characteristic (ROC) plot. The magnitude of the area that lies under the ROC plot is a measure of variation explained by the fitted multivariable logistic regression model. In this case, the area under the ROC plot is 61.00%. The unexplained proportion of variation is equal to 39.00%. Such a proportion of unexplained variation shows that the fitted model is reasonably acceptable in explaining the variability in ESBL-R *E. coli* positivity among pets as a function of the explanatory variables used for the logistic regression analysis model. Figure 1B shows a plot of sensitivity/specificity versus probability cut-off point. The two plots cross each other reasonably away from the vertical axis. This shows that the fitted model is modestly sensitive and specific.

### 3.3. Antimicrobial Resistance (AMR) Phenotypes among ESBL-R E. coli Isolates

For rectal swabs from animals confirmed as ESBL-R *E. coli*, one or more isolates (varied in colony morphology on TBX-ESBL agar supplement plates) were selected for screening their resistance against 12 agents representing 11 antimicrobial categories (Table 2). In total, we characterized 77 isolates from the 35 cats and dogs with confirmed positivity of ESBL-R *E. coli*. Ciprofloxacin resistance was evident in 57.14% (44/77) of the isolates; 38.96% (30/77) of the isolates were resistant to chloramphenicol; and 35.06% (27/77), 58.44% (45/77), 79.22% (61/77) showed resistance to gentamicin, tetracycline, and trimethoprim-sulfamethoxazole, respectively (Table 2).

None of the isolates exhibited resistance to imipenem (Table 2). The percentage of resistance among *E. coli* isolates (*n* = 77) to azithromycin, ceftriaxone, cefoxitin, and cefepime was 12.99% (10/77), 94.81% (73/77), 5.19% (4/5), and 48.05% (37/77), respectively (Table 2). PCR screening of β-lactamase genes in a subset (*n* = 50) of the confirmed ESBL-producing *E. coli* indicated the presence of the CTX-M gene in 82% (21/50) of the isolates and TEM in 50% (25/50) of the isolates. None of the isolates showed positive PCR amplification for SHV and OXA genes.

Among the characterized ESBL-producing *E. coli*, multidrug resistance phenotypes were evident in 91% (70/77) of them, ranging from carrying resistance to three and up to ten different antimicrobial categories (Table 3), the most reported resistance pattern involving four and seven antimicrobial categories, in 22.86% (16/77) for each pattern. The common multidrug resistance phenotype patterns are provided in Table 3.

## 4. Discussion

In this study, we report the findings of the first AMR carriage survey of pet cats and dogs in the UAE. The excretion of antimicrobial-resistant bacteria in the feces of domestic cats and dogs can play a role in spreading antimicrobial resistance in the environment. Additionally, the feces of these pets can serve as a potential source for humans to acquire non-pathogenic bacteria, like *E. coli*, which can be a major contributor to the spread of antimicrobial resistance genes. [11]. The evidence on AMR in animals, notably on pets, is very limited in the UAE and across the GCC countries, so our study provides an initial insight that adds to the local and regional gap in knowledge on such an important One Health topic.

The first *National Strategy and Action Plan for Combatting Antimicrobial Resistance in the UAE* was released in 2019. The first objective of such a strategy is concerned with “improving the level of public awareness regarding AMR and promoting behavioral change at the public and professional levels under the “One Health” approach” [19]. In this context, information about the frequency of AMR among companion animal *E. coli* to critically important antimicrobial (CIA) classes will be of added value to the national strategy makers. Our study highlights that ESBL-R *E. coli* was detected at a sizable rate (23.65%) among healthy pet cats and dogs sampled in the UAE, and the majority of the characterized ESBL-R *E. coli* isolates are multidrug-resistant and that involved CIA such as fluoroquinolones and 3rd and 4th generation cephalosporins. Our findings highlight the underemphasized significance of pet animals in the One Health perspective of AMR in the UAE. The treatment of infections in companion animals is similar to that in humans, and veterinarians are authorized to prescribe fluoroquinolones and cephalosporins registered for use in dogs and cats in the UAE, often without restrictions. Additionally, although it is not a widespread practice, veterinarians specializing in companion animals may choose to use human antimicrobial formulations off-label in a few exceptional situations. [20]. It is important for both pet owners and veterinarians to follow prudent use guidelines and practice hygienic measures, infection control, and antimicrobial stewardship strategies in order to reduce the potential transmission of antimicrobial-resistant bacteria in pet animal practice [21].

It may be challenging to interpret and compare the findings of this study on an international level due to variations in factors such as study design, drugs assessed, determination of breakpoints, and geographic or temporal differences. A recent study in Saudi Arabia identified ESBL-producing *E. coli* in 4.1% and 2.9% of healthy and diseased cats in a study conducted in one veterinary clinic [22]. A study in Israel concluded the prevalence of ESBL-producing *Enterobacterales* of 6.2% in healthy community dogs [23]. In Turkey, 16.8% of the *Enterobacterales* isolates from companion dogs exhibited the ESBL phenotype [24]. Other studies found ESBL-producing *E. coli* in 49 healthy dogs in Greece (20.4%) [25] and 68 dogs in Egypt (22%) [26]. In general, the isolation rate of ESBL-R *E.coli* (23.65%) in our study from the UAE is higher than that found in studies from neighboring countries.

MDR among ESBL-producing Gram-negative bacteria is considered a global health problem, and MDR phenotypes were evident in the majority (91% (70/77)) of the characterized ESBL-producing *E. coli* from cats and dogs in our study. Biologically speaking, this result was not unexpected. The ESBL enzymes are mutant, plasmid-mediated β-lactamases derived from older, broad-spectrum β-lactamase (e.g., TEM-1, TEM-2, SHV-1). Thus, they mediate resistance to extended-spectrum (third-generation) cephalosporins (e.g., ceftazidime, cefotaxime, and ceftriaxone) [27]. As reported previously, *bla_CTX-M_* genes are the most frequent ESBL-encoding genes identified in human and veterinary medicine and are often associated with *bla_TEM_* on MDR plasmids [20,22,23]. This is mirrored in our study, with the CTX-M gene present in 82% (21/50) of the ESBL-producing *E. coli* from cats and dogs in the UAE. Currently, a comparative genomics investigation is in progress to verify the phylogenetic groups, multilocus sequence types, plasmids, and resistance genes in specific MDR-ESBL-producing *E. coli* isolates from this study.

The investigation into the prevalence and risk factors of antimicrobial-resistant *E. coli* carriage in pet cats and dogs offers a means of characterizing a companion animal population that is distinct from those impacted by clinical disease [28], and can serve as a baseline for monitoring trends in AMR in this population. The questionnaires administered to the owners in the present study revealed that animal exposure to water in ditches and puddles was a risk factor associated with the carriage of ESBL-R *E. coli* in the present study population of pet dogs and cats. From observation, the ditches in the UAE urban settings are well maintained and typically are those dug for water pipeline projects. In sub-urban areas, many of the local (Emirati) families continue the pastoral way of life of their forebears by maintaining small livestock holdings (in Arabic called “izba”); in such settings, effluent/wastewater might still run through the ditches/puddles. Water sources have been recognized as a significant reservoir of antibiotics and antibiotic-resistant bacteria reviewed in [29], contributing to the transfer of resistance genes between pathogenic and non-pathogenic bacteria and potentially promoting the persistence of resistance in the environment [30].

One limitation of this study was the absence of clinical history data for the cats and dogs sampled. Moreover, the lack of specific information about previous antimicrobial usage in the dogs studied limits the ability to draw conclusions about the relationship between antimicrobial use and subsequent AMR development. Additionally, future research should aim to collect a larger number of isolates from companion animals to improve the accuracy of frequency and risk factor estimations beyond the present study’s findings. Despite these limitations, this study presents the first collection of *E. coli* isolates obtained from a population of dogs and cats in the UAE, with a sizable sample size and diverse geographical sources.

## 5. Conclusions

In this research, we present the first examination of antimicrobial resistance in *E. coli* obtained from domestic cats and dogs in the UAE. The incidence of ESBL-R *E. coli* in the animals examined in this study is significant, and due to the close bond that most owners have with their pets, these animals may pose a potential health threat to their owners and individuals with occupational exposure (e.g., veterinarians). Our findings call for strengthening the local (at Emirate level) and national (harmonized country-wide level) pet surveillance program and the implementation of antimicrobial stewardship strategies among veterinarians in the UAE to reduce the risk of transmission of ESBL-R *E. coli* among companion animals, humans, and urban environments.

## Figures and Tables

**Table 1:** Characteristics of study animals (dogs/cats combined), univariate and multivariable logistic regression model for the associations between potential risk factors and positivity of rectal swab sample to ESBL-producing *E. coli*.

Univariate Logistic Regression Analysis						
Variable	Category	Total (Missing)	Positive for ESBL-R *E. coli*	%	Odds Ratio (95% Confidence Interval)	*p*-Value ^1^
Clinic	A	46	13	28.26	Ref	
	B	40	11	27.50	0.96 (0.37–2.4)	0.937
	C	37	5	13.51	0.39 (0.12–1.2)	**0.112**
	D	21	5	23.81	0.79 (0.24–2.6)	0.703
	E	4	1	25.00	0.84 (0.08–8.8)	0.889
Species	Cat	122	32	26.23	Ref	
	Dog	26	3	11.54	0.36 (0.10–1.30)	**0.121**
Age	Less than 1 year	48	13	27.08	Ref	
	≥1 year and <2 years	37	8	21.62	0.74 (0.27–2.03)	0.563
	≥2 years and <3 years	30	8	26.67	0.97 (0.34–2.74)	0.968
	≥3 year	16	2	12.50	0.38 (0.07–1.92)	0.245
Sex	Female	58	13	22.41	Ref	
	Male	82	19	23.17	1.04 (0.46–2.32)	0.916
Frequency per week that the animal spent in the house or outside						
	Always inside	58	10	17.24	Ref	
	Always outside	13	6	46.15	4.11 (1.13–14.88)	**0.031**
	Mostly in the house	48	8	16.67	0.96 (0.34–2.66)	0.937
	Mostly outside the house	9	4	44.44	3.84 (0.87–16.88)	**0.075**
The number of other animals kept in the household						
	0	21	7	33.33	Ref	
	5	6	2	33.33	1 (0.14–6.85)	1.000
	4	14	0	-	Omitted	
	>5	48	10	29.83	0.52 (0.16–1.65)	0.271
	1	11	2	18.18	0.44 (0.74–2.63)	0.372
	3	12	3	25.00	0.66 (0.13–3.27)	0.617
	2	16	4	25.00	0.66 (0.15–2.84)	0.584
If the dog/cat stayed in a pension/animal shelter in the past year						
	No	60	14	23.22	Ref	
	Yes	67	13	19.40	0.79 (0.33–1.85)	0.589
Frequent contact with farm or livestock animals						
	Never	65	11	16.92	Ref	
	Frequently	7	3	42.86	3.68 (0.72–18.81)	**0.117**
	Rare	50	10	20.00	1.22 (0.47–3.17)	0.672
Whether dog/cat had access to open water sources in the last six months						
	No access	103	17	16.50	Ref	
	Lakes, rivers, creeks	3	2	66.67	10.11 (0.86–117.97)	**0.065**
	Water in ditches, puddles	18	8	44.44	4.04 (1.39–11.74)	**0.010**
	Toilet	2	2	100.00	Omitted	
Type of food that the owner provides to dog/cat						
	Dry food, prepacked (commercial)	66	12	18.18	Ref	
	Dry and wet foods, prepacked (commercial)	39	7	17.95	0.98 (0.35–2.75)	0.976
	Diet includes raw meat	15	6	40.00	3 (0.89–10.03)	**0.075**
	Diet included food remaining from the table	8	3	37.50	2.7 (0.56–12.87)	0.213
Multivariable Logistic Regression Analysis						
Variable—dog/cat had access to open water sources in the last six months:					Odds Ratio (95% Confidence Interval)	*p*-value
Water in ditches, puddles (vs. no access)					3.71 (1.23–11.21)	0.020

^1^ In bold, variables with a *p*-value < 0.2 those were retained from the univariate analysis to be included in the multivariable model.

**Table 2:** Antimicrobial resistance phenotypes of ESBL-R *E. coli* isolates (*n* = 77) from rectal swabs of domestic pets (cats and dogs) in the United Arab Emirates.

Antimicrobial Category	Antimicrobial Agent	No. of ESBL-R *E. coli* Isolates (*n* = 77)		
		Resistant *n* (%)	Intermediate *n* (%)	Susceptible *n* (%)
Fluoroquinolone	Ciprofloxacin (CIP)	44 (57.14)	32 (41.56)	1 (1.30)
Phenicol	Chloramphenicol (C)	30 (38.96)	1 (1.30)	46 (59.74)
Aminoglycosides	Gentamicin (CN)	27 (35.06)	—	50 (64.94)
Tetracyclines	Tetracycline (TET)	45 (58.44)	9 (11.69)	23 (29.87)
2nd generation Cephalosporins	Cefoxitin (FOX)	4 (5.19)	4 (5.19)	69 (89.61)
3rd generation Cephalosporins	Cefotaxime (CTX)	70 (90.91)	2 (2.60)	5 (6.49)
	Ceftriaxone (CRO)	73 (94.81)	1 (1.30)	3 (3.90)
4th generation Cephalosporins	Cefepime (FEP)	37 (48.05)	32 (41.56)	8 (10.39)
Carbapenems	Imipenem (IPM)	—	74 (96.10)	3 (3.90)
Sulfonamides	Trimethoprim-sulfamethoxazole (SXT)	61 (79.22)	—	16 (20.78)
Penicillin	Ampicillin (AMP)	75 (97.40)	—	2 (2.60)
Macrolides	Azithromycin (AZM)	10 (12.99)	—	67 (87.01)

**Table 3:** Pattern of resistance phenotypes found in multidrug resistance ESBL-R *E. coli* isolates (*n* = 70) from rectal swabs of domestic pets (cats and dogs) in the United Arab Emirates.

No. Antimicrobial Category	No. of Isolates (%)	Common Resistance Patterns * (No. of Isolates)
3	7 (10.00)	3rdGC-S-P (4)
4	16 (22.86)	3rdGC-4thGC-S-P (6) FQ-3rdGC-S-P (4)
5	9 (12.86)	FQ-T-3rdGC-S-P (4)
6	15 (21.43)	FQ-Ph-3rdGC-4thGC-S-P (7)
7	16 (22.86)	FQ-Ph-A-T-3rdGC-S-P (7) A-Ph-T-3rdGC-4thGC-S-P (4) FQ-A-T-3rdGC-4thGC-S-P (4)
8	3 (4.29)	FQ-Ph-A-T-3rdGC-4thGC-S-P (3)
9	3 (4.29)	FQ-Ph-A-T-3rdGC-4thGC-S-P-M (3)
10	1 (1.43)	FQ-Ph-A-T-2ndGC-3rdGC-4thGC-S-P-M
Total	70 (100.00)	

* Fluoroquinolone, FQ; Phenicol, Ph; Aminoglycosides, A; Tetracyclines, T; 2nd generation Cephalosporins, 2ndGC; 3rd generation Cephalosporins, 3rdGC; 4th generation Cephalosporins, 4thGC; Sulfonamides, S; Penicillins, P; Macrolides, M.

## Data Availability

The data presented in this study are available on request from the corresponding author. The data are not publicly available due to ethical restrictions.
